# Peritoneal Dialysis in Infant Methylmalonic Acidemia: Effective Management Despite Sterile Peritonitis

**DOI:** 10.7759/cureus.111554

**Published:** 2026-06-26

**Authors:** Mustakiz Z Manir, Utsav Haldar, Niranjan Gopal, Palani Selvam Mohanraj, Meenakshi Girish, Divya Dhawan

**Affiliations:** 1 Biochemistry, All India Institute of Medical Sciences, Nagpur, Nagpur, IND; 2 Medical Biochemistry, All India Institute of Medical Sciences, Nagpur, Nagpur, IND; 3 Biochemistry, All India Institute of Medical Sciences, Gorakhpur, Gorakhpur, IND; 4 Pediatrics, All India Institute of Medical Sciences, Nagpur, Nagpur, IND

**Keywords:** hyperammonemia, infant, metabolic crisis, methylmalonic acidemia, peritoneal dialysis

## Abstract

Methylmalonic acidemia (MMA) is one of the rare inherited metabolic disorders, and the affected patients usually suffer from repeated life-threatening metabolic crises in which hyperammonemia and acidosis are observed. Although continuous venovenous hemodialysis is considered the first choice for extracorporeal detoxification in neonates and infants, the available experience regarding peritoneal dialysis in this group of patients is still limited, and how to optimally manage the complications of dialysis has not been clearly described. We report the case of a three-month-old female infant with isolated MMA who was brought in with vomiting for four days, cough for seven days, and fever for one day. Even after she was started on sodium benzoate, L-carnitine, and sodium bicarbonate, encephalopathy gradually became worse, plasma ammonia level increased from 188 μmol/L to 332 μmol/L, and severe metabolic acidosis was observed (bicarbonate: 9 mEq/L). Peritoneal dialysis was then started, and the metabolic response was found to be excellent: ammonia level came down to 86 μmol/L after eight cycles of dialysis and further to 61 μmol/L on hospital day four. After completion of 17 cycles of dialysis, culture-negative peritonitis was noted in the patient (150 cells/mm³ with 57% neutrophils), and this was managed by escalation of antibiotics together with removal of the catheter. The infant was finally discharged on day 23 in stable condition, with the ammonia level back to normal (49 μmol/L) and the metabolic acidosis showing resolution. From the present case, it can be stated that peritoneal dialysis is effective for the management of severe hyperammonemia in infants suffering from MMA, and rapid correction of the metabolic disturbance was achieved even though the course was complicated by peritonitis. The outcome obtained in our patient supports the expert consensus that, in this age group, peritoneal dialysis can be used as a reasonable alternative to continuous renal replacement therapy, especially at centers where pediatric peritoneal dialysis experience is available.

## Introduction

Methylmalonic acidemia (MMA) is inherited in an autosomal recessive pattern and occurs because of either a deficiency of methylmalonyl-coenzyme A mutase or defects in the synthesis of its cofactor from vitamin B12 [1,2]. MMA is a common organic acidemia with an incidence of 1.14 per 100,000 live births [1]. Infants with severe MMA present early in life with metabolic decompensation, showing metabolic acidosis, hyperammonemia, ketosis, and encephalopathy [1,2]. The management of acute metabolic decompensation in MMA requires intensive care, including protein restriction, fluid resuscitation, sodium bicarbonate for acidosis correction, and ammonia-lowering therapies [1,2]. When medical management fails, particularly during severe hyperammonemia, extracorporeal detoxification becomes necessary [1,3]. While continuous renal replacement therapy (CRRT) is preferred for neonates due to its excellent clearance [3,4], peritoneal dialysis is a viable alternative, especially in centers with established pediatric peritoneal dialysis programs [3]. European Reference Networks guidelines note the effectiveness of peritoneal dialysis in infants due to their large peritoneal surface area relative to body size [3]. We present the case of a three-month-old female infant with MMA who developed severe metabolic acidosis and hyperammonemia requiring peritoneal dialysis, with successful management of the subsequent sterile peritonitis. Therefore, this case highlights the practical role of peritoneal dialysis as an effective alternative for severe hyperammonemia in infantile MMA, especially when CRRT is not readily available. It also emphasizes the need for early extracorporeal detoxification when medical therapy fails and close monitoring for dialysis-related complications such as sterile peritonitis.

## Case presentation

A three-month-old female infant with MMA was admitted with four days of vomiting, seven days of cough, and one day of fever. The infant was born at term via cesarean section, weighing 2.25 kg, and cried after birth. Family history revealed the death of an elder sibling, suggesting an inherited metabolic disease. Diagnostic tests showed elevated methylmalonic acid levels in the urine, and magnetic resonance spectroscopy showed a lipid-lactate peak, indicating mitochondrial dysfunction. The child was healthy until one week prior, when she developed a productive cough. Four days before presentation, she experienced three to four daily episodes of non-bilious vomiting containing food. She developed intermittent fever one day before admission, which responded to antipyretics. Although she initially maintained normal urine output and oral intake, she became progressively lethargic over the 24 hours before admission.

Physical examination showed a lethargic but arousable infant with stable vitals: afebrile, heart rate of 130 beats/minute, blood pressure of 82/39 mmHg, respiratory rate of 27 breaths/minute, and oxygen saturation of 98% on room air. The anterior fontanelle was level and non-pulsatile. Respiratory examination revealed equal air entry and wheezing. Neurological examination showed only lethargy without focal deficits. Laboratory investigations revealed severe hyperammonemia and metabolic acidosis. Plasma ammonia level was 188 µmol/L on day one, rising to 332 µmol/L by day three. Serum bicarbonate was 9 mEq/L, indicating metabolic acidosis. Serum lactate was elevated. Renal function showed normal creatinine with low blood urea nitrogen. Hepatic transaminases were mildly elevated (aspartate aminotransferase: 27 U/L and alanine aminotransferase: 33 U/L). Serum albumin was found to be 3.5 g/dL, and C-reactive protein was <5.0 mg/L. On complete blood count, hemoglobin, white cell count, and platelets were all within normal limits. Cultures of both blood and urine returned negative (Table 1). Due to rising ammonia levels, worsening acidosis, and encephalopathy despite conservative management, the patient was made nil per os and started on intravenous dextrose fluids to suppress protein catabolism. For severe metabolic acidosis, intravenous sodium benzoate was given. As the clinical condition continued to worsen, with encephalopathy becoming deeper and the ammonia level reaching 332 µmol/L, the nephrology team was consulted, and after considering the age of the infant, the severity of hyperammonemia, and the institutional expertise available, the decision was taken to start peritoneal dialysis.

**Table 1:** Laboratory and diagnostic findings supporting methylmalonic acidemia with sterile peritonitis. AST: aspartate aminotransferase; ALT: alanine aminotransferase; CRP: C-reactive protein; MRS: magnetic resonance spectroscopy

Investigation	Patient result	Reference range/Expected finding	Clinical significance
Urinary methylmalonic acid	Elevated	Not elevated/absent in healthy individuals	Supports the diagnosis of methylmalonic acidemia
Magnetic resonance spectroscopy	Lipid-lactate peak present	No lipid-lactate peak expected	Suggestive of metabolic injury/mitochondrial dysfunction
Plasma ammonia on admission	188 µmol/L	11–50 µmol/L	Hyperammonemia
Peak plasma ammonia	332 µmol/L	11–50 µmol/L	Severe hyperammonemia requiring urgent intervention
Plasma ammonia after treatment	49 µmol/L	11–50 µmol/L	Improvement after treatment and peritoneal dialysis
Serum bicarbonate	9 mEq/L	22–28 mEq/L	Severe metabolic acidosis
Serum lactate on admission	1.7 mmol/L	0.7–2.1 mmol/L	Within the reference range initially
Peak serum lactate	7.9 mmol/L	0.7–2.1 mmol/L	Lactic acidosis during the hospital course
Serum lactate near discharge	4.6 mmol/L	0.7–2.1 mmol/L	Persistent but improving hyperlactatemia
Blood urea	6 mg/dL	15–39 mg/dL	Low blood urea level
Serum creatinine	0.4 mg/dL	<0.90 mg/dL	No evidence of renal impairment; low value likely age-related
Aspartate aminotransferase	27 U/L	0–45 U/L	Within the reference range initially
Alanine aminotransferase	33 U/L	0–45 U/L	Within the reference range initially
Peak aspartate aminotransferase	73 U/L	0–45 U/L	Mild transaminitis during the hospital course
Peak alanine aminotransferase	51 U/L	0–45 U/L	Mild transaminitis during the hospital course
Serum albumin on admission	3.5 g/dL	3.2–4.5 g/dL	Within the reference range initially
Lowest serum albumin	<1.0 g/dL	3.2–4.5 g/dL	Marked hypoalbuminemia; clinical correlation required
Total protein on admission	5.2 g/dL	6.4–8.3 g/dL	Hypoproteinemia
Lowest total protein	<2.0 g/dL	6.4–8.3 g/dL	Marked hypoproteinemia; clinical correlation required
C-reactive protein on admission	<5.0 mg/L	<5.0 mg/L	No significant inflammatory response initially
Peak C-reactive protein	34.3 mg/L	<5.0 mg/L	Raised inflammatory marker during the hospital course
Peritoneal fluid appearance	Pale yellow, clear fluid	Clear, non-purulent fluid expected	Non-purulent gross appearance
Peritoneal fluid total cell count	150 cells/mm³	No significant inflammatory cells expected	Mild inflammatory cellular response
Peritoneal fluid differential count	Neutrophils 57%, lymphocytes 43%	No significant neutrophilic predominance expected	Supports inflammatory peritoneal fluid changes
Repeat peritoneal fluid total cell count	100 cells/ mm³	No significant inflammatory cells expected	Persistent mild inflammatory response
Repeat peritoneal fluid differential count	Neutrophils 60%, lymphocytes 40%	No significant neutrophilic predominance expected	Consistent with peritoneal inflammation
Peritoneal fluid sugar	168 mg/dL	Interpret with simultaneous blood glucose	No isolated diagnostic significance without paired blood glucose
Peritoneal fluid total protein	<2.0 g/dL	Laboratory reference not specified	Low-protein peritoneal fluid
Peritoneal fluid Gram stain	Rare pus cells seen; no organism seen	No organism seen	Inflammatory cells without an identifiable organism
Peritoneal fluid culture	No growth after 48 hours of aerobic incubation	No growth expected	Supports the absence of bacterial growth
Peritoneal fluid cytology	Few mature lymphocytes and occasional neutrophils; negative for malignant cells	No malignant cells expected	Supports sterile inflammatory peritonitis
Blood culture	No growth reported	No growth expected	No documented bloodstream bacterial growth
Urine routine microscopy	Trace ketone; no protein, blood, glucose, nitrite, leukocytes, pus cells, red blood cells, casts, crystals, or bacteria	No ketone or abnormal urinary cells expected	Trace ketonuria; otherwise unremarkable urine microscopy
Peripheral smear	Normocytic normochromic red blood cells; adequate platelets; no malarial parasite seen	No hemoparasite expected	No malarial parasite detected
Human immunodeficiency virus antibody test	Non-reactive	Non-reactive	Negative screening test
Hepatitis B surface antigen	Non-reactive	Non-reactive	Negative screening test
Anti-hepatitis C virus antibody	Non-reactive	Non-reactive	Negative screening test

The patient was shifted to the pediatric intensive care unit on day two. A catheter for peritoneal dialysis was placed, and dialysis was started with rapid exchanges of short dwell time. As per the prescription, 17 cycles were planned with standard dialysis solution. After eight cycles, ammonia decreased to 86 µmol/L by June 12 and 61 µmol/L by June 13. The level of consciousness of the patient gradually improved, and the metabolic acidosis resolved. Serum lactate value came down from 4.5 to 2.5 mmol/L. L-carnitine was started at a dose of 200 mg/kg/day to help in the excretion of toxic metabolites, while vitamin B12 supplementation was continued as before. Feeds through the enteral route were restarted using a low-protein formula along with breast milk to fulfill the nutritional requirements. On days five to six, after completion of all 17 cycles of peritoneal dialysis, fever appeared in the patient, even though broad-spectrum antibiotics were already on board. Analysis of peritoneal fluid revealed 150 cells/mm³, which raised the suspicion of peritoneal dialysis-associated peritonitis. Samples of the fluid were then sent for Gram staining, culture, and cytological examination. Glucose was 168 mg/dL, and protein was <2.0 g/dL. On Gram staining, only rare pus cells were seen, and no organism was identified. Following discussion with the nephrology team, the antibiotics were escalated, and peritoneal dialysis was discontinued. To avoid the risk of persistent infection, the dialysis catheter was also removed. After 48 hours, cultures of the peritoneal fluid came back negative, and on cytology, only a few scattered lymphocytes and neutrophils were seen, which was suggestive of sterile peritonitis. Oral antibiotics were continued even after the catheter was removed. On repeat analysis done on day seven, the cell count had reduced to 100 cells/mm³, and no pus cells or organisms could be detected on Gram stain.

Some minor complications were noted during the course of the hospital stay. Regurgitation was managed by giving frequent small feeds along with domperidone. On days 10-11, ammonia levels were again raised to the range of 210-244 µmol/L, for which sodium benzoate and L-arginine had to be administered. Sodium bicarbonate was also administered for control of the metabolic acidosis. Loose stools, when present, were managed with probiotics and zinc supplementation. With continued monitoring, ammonia, lactate, and blood gas parameters were observed to return to normal levels. At the time of discharge on day 23, ammonia level was 49 µmol/L, lactate was 4.6 mmol/L, and the acidosis was showing further improvement.

On discharge, the medications prescribed to the patient were sodium benzoate, L-carnitine, vitamin B12, L-arginine, sodium bicarbonate, and antibiotics for peritonitis. For nutrition, a special metabolic formula (MMA feed) was advised in combination with breast milk/infant formula. Counseling was provided to the family regarding management of MMA, the need for protein restriction, and how to recognize early signs of metabolic decompensation. A follow-up visit was planned after three days, at which time plasma ammonia, lactate, and blood gas were to be checked again. Table 2 presents a summary of the biochemical and clinical course during hospitalization.

**Table 2:** Serial biochemical and clinical course during hospitalization. Serial bicarbonate and complete blood gas values were not available for all time points in the discharge summary; therefore, only the documented bicarbonate value and recorded clinical acid–base status are presented. PD: peritoneal dialysis; NPO: nil per oral; HCO₃⁻: bicarbonate

Clinical phase	Plasma ammonia	Serum lactate	Acid–base status	Neurological status	Intervention/Outcome
Initial metabolic crisis	188 µmol/L	Not available	Metabolic acidosis documented later; HCO₃⁻ 9 mEq/L	Lethargic but vitally stable	NPO, dextrose IV fluids, sodium benzoate started
Clinical worsening before dialysis	332 µmol/L	Not available	Severe metabolic acidosis; HCO₃⁻ 9 mEq/L	Worsening encephalopathy	Nephrology opinion taken; peritoneal dialysis planned
After 8 rapid PD dwells	86 µmol/L	3.5 mmol/L	Acidosis improving	Sensorium improved	Good initial biochemical response to PD
Early post-PD response	61 µmol/L	2.5 mmol/L	Metabolic acidosis improving/resolving	Clinically improved	Continued monitoring and supportive therapy
Post-dialysis stabilization	61–63 µmol/L	3.8 mmol/L	Acidosis clinically improved	Stable/Improving	Sodium benzoate continued; carnitine and vitamin B12 added
After PD completion	49 µmol/L	2.0 mmol/L	Improved acid–base status	Improved sensorium	Feeds gradually reintroduced
Monitoring phase	74 µmol/L	2.6 mmol/L	Not specifically documented	Clinically monitored	Continued conservative management
Rebound hyperammonemia	210 µmol/L	5.0 mmol/L	Worsening metabolic acidosis noted	Clinically monitored	Sodium benzoate increased; L-arginine and oral sodium bicarbonate started
Persistent rebound	244 µmol/L	6.8 mmol/L	Metabolic acidosis under treatment	Clinically monitored	Continued ammonia-lowering therapy and bicarbonate
Improving trend	94 µmol/L	7.9 mmol/L	Resolving metabolic acidosis	Stable	Continued monitoring
Recovery phase	78 µmol/L	7.9 mmol/L	Resolving metabolic acidosis	Stable	Supportive care continued
Pre-discharge phase	96 µmol/L	7.5 mmol/L	Resolving metabolic acidosis	Hemodynamically stable	Discharge planning with follow-up advice
Near discharge	49 µmol/L	4.6 mmol/L	Resolving metabolic acidosis	Hemodynamically stable	Continued oral medications and metabolic feeds

## Discussion

The present case highlights some of the important principles that have to be followed for managing MMA presenting with metabolic decompensation in early infancy. Within 48 hours, the condition of the patient worsened, plasma ammonia rose from 188 μmol/L to 332 μmol/L despite ongoing treatment, and metabolic acidosis (serum bicarbonate: 9 mEq/L) along with raised lactate (3.5-4.5 mmol/L) was also documented. Although such findings are typical of an acute metabolic crisis in isolated MMA, the successful outcome obtained in our case using peritoneal dialysis, even with the development of sterile peritonitis later on, contributes additional information to the limited literature on dialysis in infants with metabolic disease [1,2]. Hyperammonemia in MMA is mainly related to impaired urea-cycle function caused by accumulated organic acid metabolites [1,2]. Clinically, this is important because rising ammonia levels may rapidly worsen encephalopathy [5]. Therefore, persistent hyperammonemia despite medical therapy should prompt early consideration of extracorporeal detoxification [1-3].

The stepwise management followed in our case is in accordance with the European guidelines, with due emphasis on clinical judgment. As an initial step, protein intake was stopped, intravenous dextrose fluids were given to suppress protein catabolism, and sodium bicarbonate was added for correction of acidosis. Sodium benzoate (loading dose of 250 mg/kg, followed by 250 mg/kg/day) helps in reducing ammonia by allowing its conjugation with glycine, which is then excreted in the urine. L-carnitine, when given at 200 mg/kg/day, helps in the excretion of toxic metabolites and makes up for the loss of carnitine. As the medical measures could not stop further deterioration and ammonia levels remained high, peritoneal dialysis was initiated in view of the severe encephalopathy along with ammonia levels in the range of 250-500 μmol/L [1,2].

Selection between peritoneal dialysis and continuous venovenous hemodialysis (CVVHD) needs careful consideration, as guidelines mention CVVHD as the preferred option for neonates with MMA [1-2]. However, in the 2024 European Reference Network consensus, it is acknowledged that "peritoneal dialysis represents a good option in infants" and "has good effectiveness on plasma methylmalonic acid clearance, due to their large peritoneum proportional to body weight" [3]. In the present case, peritoneal dialysis was found to be effective in bringing down ammonia from 332 μmol/L to 86 μmol/L after eight cycles, and further to 61 μmol/L by day four. Sterile peritonitis after cycles of peritoneal dialysis is a known complication, although the exact incidence in infants suffering from metabolic disease has not been well documented [1,3]. The absence of organisms on Gram stain, together with negative bacterial cultures, pointed toward sterile peritonitis, which may occur due to chemical irritation, biofilm infection, or bacterial translocation [4].

The favorable outcome obtained in this patient, who achieved hemodynamic stability and normalization of the ammonia level (49 μmol/L) by hospital day 23, is in contrast with the outcomes reported in the pre-modern intensive care era. The long-term outcome is still not known, but rapid metabolic correction by modern peritoneal dialysis may help in reducing neurologic injury caused by hyperammonemia, as the prognosis depends mainly on the peak ammonia level and the duration of crisis [1,2]. Long-term follow-up under nephrology is also very important because chronic kidney disease eventually develops in 47-66% of patients with isolated MMA, with mut⁰ and cblB genotypes carrying the highest risk (61-66%), and the median age at onset of chronic kidney disease being 8-13.5 years [3,6]. For monitoring of kidney function, glomerular filtration rate estimates based on cystatin C should be preferred, because formulas based on creatinine tend to overestimate kidney function by 16-37 mL/minute/1.73 m² in these children, owing to their reduced muscle mass [3,6].

There are some limitations of this case report that need to be acknowledged. As this is a report of a single case, the findings may not be generalizable to all patients, and in the absence of comparative data, it is not possible to draw a definitive conclusion regarding the superiority of peritoneal dialysis. The choice of dialysis modality was guided by institutional expertise rather than by randomized allocation, and therefore, some selection bias is also present. Even so, the present case is of importance because it documents the modern use of peritoneal dialysis in a three-month-old infant with severe MMA-related hyperammonemia, an area where the available evidence is still limited.

## Conclusions

In our patient, peritoneal dialysis was found to be effective for the management of severe hyperammonemia in an infant with MMA, with rapid correction of the metabolic disturbance. It is suggested that clinicians should start extracorporeal detoxification once ammonia levels go beyond 250 μmol/L, along with clinical deterioration. CVVHD is the preferred modality, but peritoneal dialysis may be considered a reasonable alternative in selected infants, particularly when CRRT is unavailable or not feasible, mainly because infants have a relatively larger peritoneal surface area. Long-term follow-up by metabolic specialists, nephrologists, and developmental pediatricians is also essential because of the high risk of chronic kidney disease, neurodevelopmental impairment, and recurrent metabolic decompensations. Systematic reporting of outcomes of peritoneal dialysis in infant metabolic disease, including both successful cases and complications, will help in further refinement of the management protocols.
